# Physiotherapy Intervention for Grade 4 Rheumatoid Arthritis Managed With Total Knee Replacement: A Case Report

**DOI:** 10.7759/cureus.46658

**Published:** 2023-10-07

**Authors:** Shraddha S Kochar, Mitushi Deshmukh, Pranali S Fokmare

**Affiliations:** 1 Neurophysiotherapy, Ravi Nair Physiotherapy College, Datta Meghe Institute of Higher Education and Research, Wardha, IND; 2 Musculoskeletal Physiotherapy, Ravi Nair Physiotherapy College, Datta Meghe Institute of Higher Education and Research, Wardha, IND

**Keywords:** exercises, rehabilitation, physical therapy, total knee replacement, rheumatoid arthritis

## Abstract

Inflammatory arthritis and extra-articular involvement remain characteristic features of the systemic autoimmune disease rheumatoid arthritis (RA). Metatarsophalangeal joints, wrists, shoulders, elbows, hips, knees, and ankles are among the joints that are most commonly infected. The patient in this case report is a 30-year-old woman with a history of deformities in both hands and legs. She approached the hospital for an examination, where she was confirmed with grade 4 RA. As the condition was severe in the bilateral knee joint, she was first managed with total knee replacement of the left side. Due to this, there was pain around the left knee joint, and also, there was a restriction in the range of motion of the knee joint; for this, the patient was advised for physiotherapy. The patient was regularly treated for 15 days. We report that physical therapy following the surgery significantly reduced pain intensity, improved the knee joint's range of motion, and increased the strength of the muscles around the knee.

## Introduction

As with any chronic disease, rheumatoid arthritis (RA) arises in stages, beginning with an asymptomatic pre-rheumatoid arthritis stage to officially diagnosed RA as well as late/refractory RA, and the causes and consequences of each of these stages continue to be far away from being totally understood, and the contributing factors causing a switch from one stage to the subsequent one are yet to be fully realized [1]. RA is a condition indicated by inflammatory changes that occur in the synovial tissue of joints, even bone and cartilage, and, fewer times, in extra-articular areas, and the precise cause of RA is obscure [2]. Long before clinically noticeable arthritis shows up, the progression of the illness begins as an immune system disorder that remains asymptomatic and evolves through quite a few stages till the condition is eventually diagnosed as RA [3]. Currently, the categorization of RA is centered on an array of usual laboratory and clinical results, incorporating findings of abnormalities that have damaged organ systems and anatomical structures; the emergence of presumed etiological mechanisms; genetic factors; and, on occasion, infectious agents; and also the overall features associated with clinical manifestations of the autoimmune condition [4]. The HLA-DRB1 gene, which encodes the HLA-DRB1 molecule along with one of the particular alleles of this gene that have been correlated with RA, encodes a conserved sequence of amino acids that resides in the antigen binding groove of the antigen-presenting molecule and this gene is considered to be the largest genetic risk indicator for RA [5]. Considering early diagnosis may prevent the further development of the disease in several people, eliminating or greatly postponing it, incurable joint damage, and disability in as many as ninety percent of RA patients, an accurate and timely diagnosis is of the greatest significance in the treatment of RA [6].

The knee joint tends to be associated with people with RA as the disease advances [7]. Within the first five years shortly after receiving their initial diagnosis, 17 percent of people diagnosed with RA undergo an orthopedic procedure, while more than one-third of those diagnosed are going to require a major joint replacement, the majority of which will entail a total knee or hip replacement (TKR and THR) [8]. TKA has been indicated to represent the best possible intervention that lowers knee pain and raises physical function in RA individuals with chronic arthritis when synovectomy offers no use; however, as RA individuals carry a higher risk for late issues, a lot of important issues about before surgery or surgical techniques should be taken into account with the goal improve the outcomes of TKA in such group of patients [9]. Treatments can be complicated with big joints' tendency for contractures, fixed flexion and valgus abnormalities, and ligamentous laxity [10]. The most effective technique to cope with RA fatigue, which is likely tricky and multivariate, is probably to cure it as a different symptom by applying an approach with multiple components involving all non-pharmacological and pharmaceutical methods [11]. Physical therapy, surgery, occupational therapy, and rest can all be advantageous [12].

## Case presentation

Patient information

The patient in this report is a 30-year-old woman who works as a homemaker and has right-handed dominance. She had been fine two years back when she started developing a bilateral deformity, initially in her hands and then in her feet. The deformity was insidious in onset and gradually progressive, with pain in bilateral upper and lower limbs, particularly in both knees. The pain grew worse over two years to the point where the patient cannot perform her daily living activities and also has difficulty walking. She additionally shared a history of whole-body stiffness. She previously visited a private hospital with this issue, where analgesics were prescribed to assist her with the pain. However, since she received only symptomatic alleviation, she visited the orthopedic outpatient department with complaints of bilateral upper and lower limb pain, with the knee more involved. An X-ray and blood test were carried out in this location. In both knee joints, the X-ray showed grade 4 RA. On blood test, the RA qualitative test showed 44.6 U/mL which is very high and suggestive of RA. She was on oral medications for RA but as the condition got worse, she was then advised to proceed with the left knee's total replacement. She has no history of trauma or falls and has regular bowel and bladder functions. After the surgical procedure, the patient's main complaints were pain around the knee joint and a restriction in the knee joint movement; therefore, the patient was referred to musculoskeletal physiotherapy for further management.

Clinical findings

The patient's written and verbal consent was acquired prior to conducting the physical examination. On observation, the patient was a mesomorph, lying supine, and hemodynamically stable. There were visible deformities seen in the fingers and foot of both the hands and leg, like Boutonniere deformity of both thumbs, fingers showing swan neck deformity and ulnar deviation of metacarpophalangeal joints, and hallux valgus left great toe, and clawing of toes of both the foot and supinated foot, respectively. Movement restrictions were present at small joints of the finger and foot bilaterally. Before the operation, crepitus was felt bilaterally in the knee joint, with the active range being 20-55 degrees on the right and 20-50 degrees on the left knee. Fixed flexion deformity of the right lower limb and slight extension lag of the left knee can be seen.

**Figure 1:**
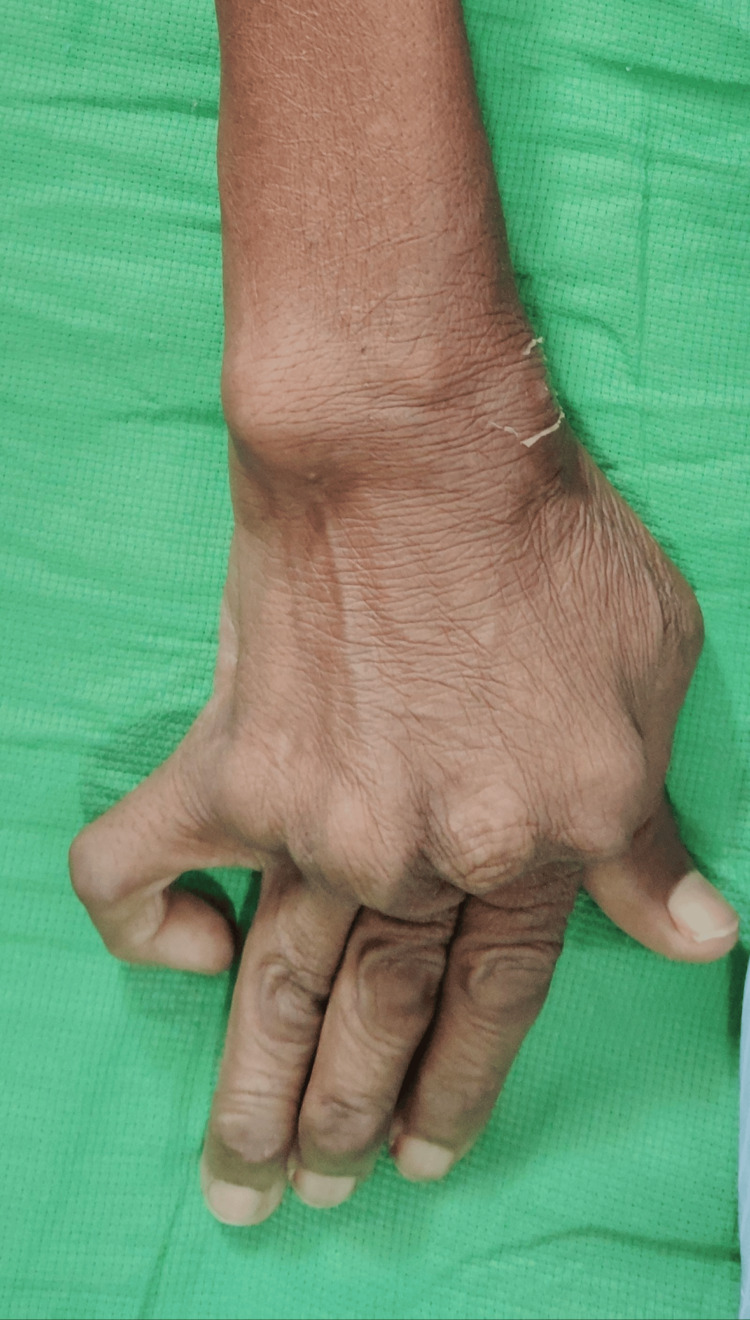
Deformities in the left hand.

**Figure 2:**
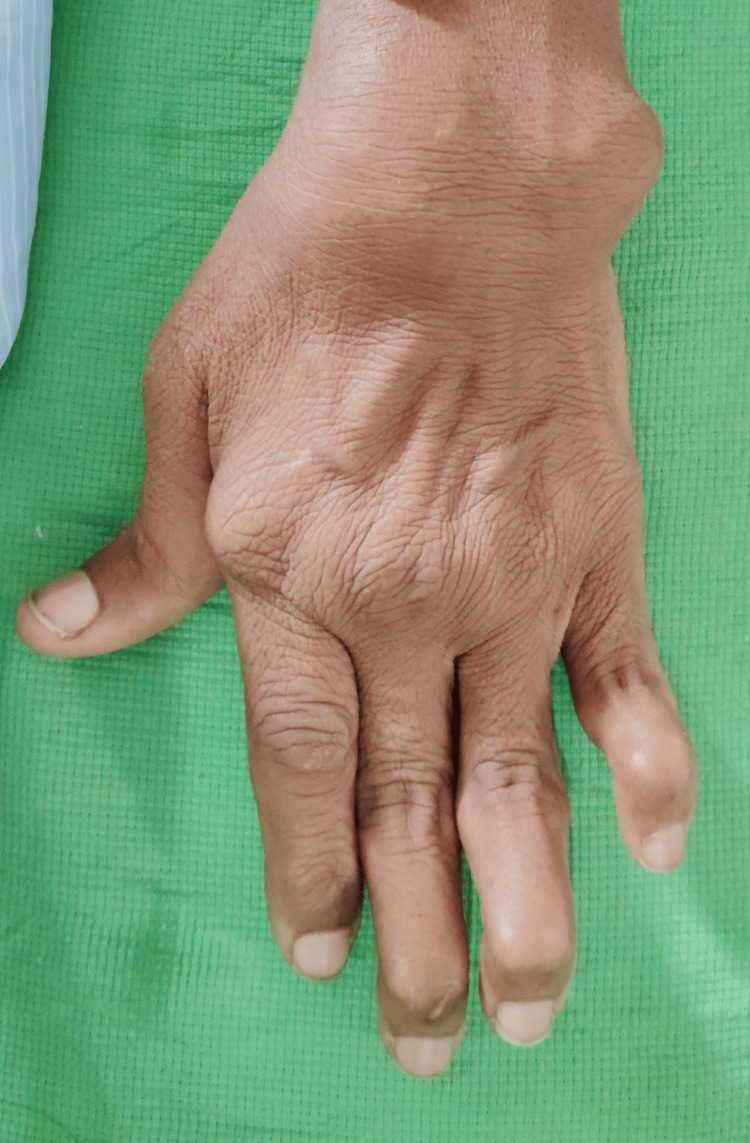
Deformities in the right hand.

**Figure 3:**
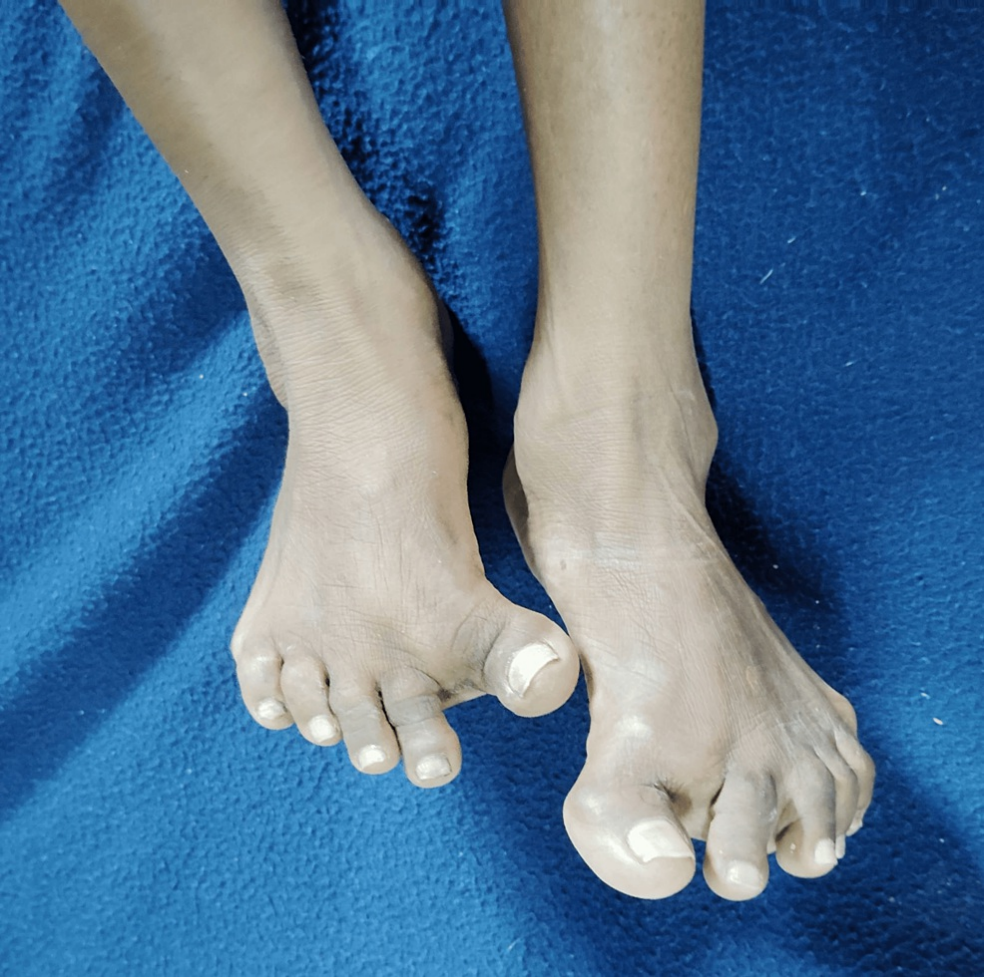
Deformities in both the lower limbs.

**Figure 4:**
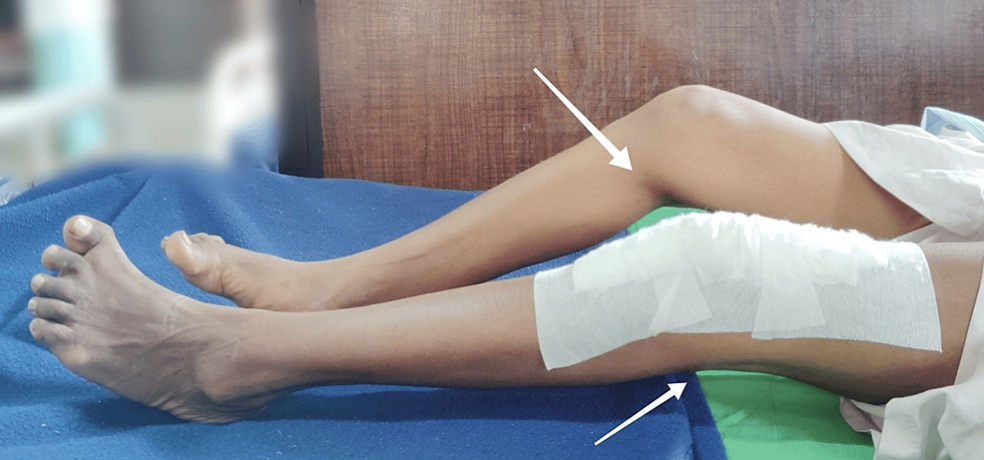
Fixed flexion deformity of knee joints

On examination of the operated site, no swelling was present; the incision length was approximately 12 cm. On palpation, there is a restriction of the knee joint range of motion (ROM); tenderness is grade 2. The patient rates the intensity of pain prior to the operation on the visual analog scale as 7.5/10 on activity and 2.1/10 on rest (Figures 1-4).

Radiological investigations

**Figure 5:**
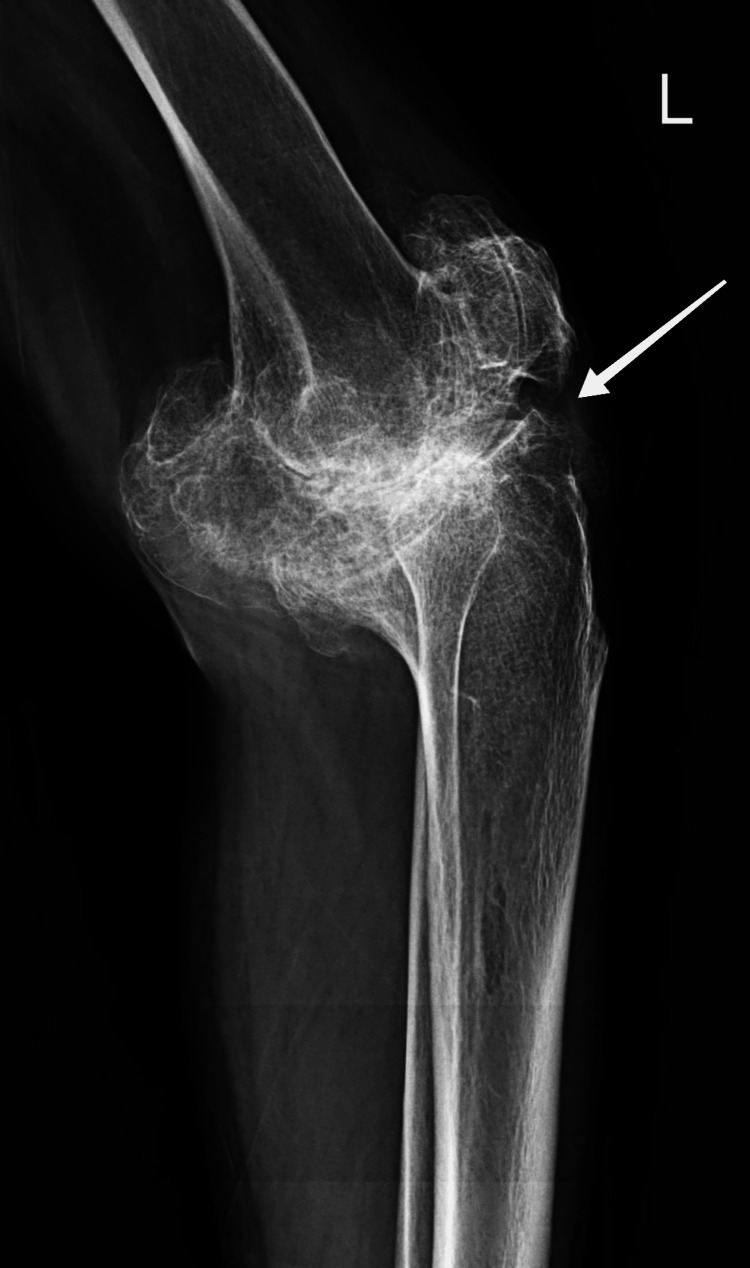
Pre-operative left knee X-ray.

**Figure 6:**
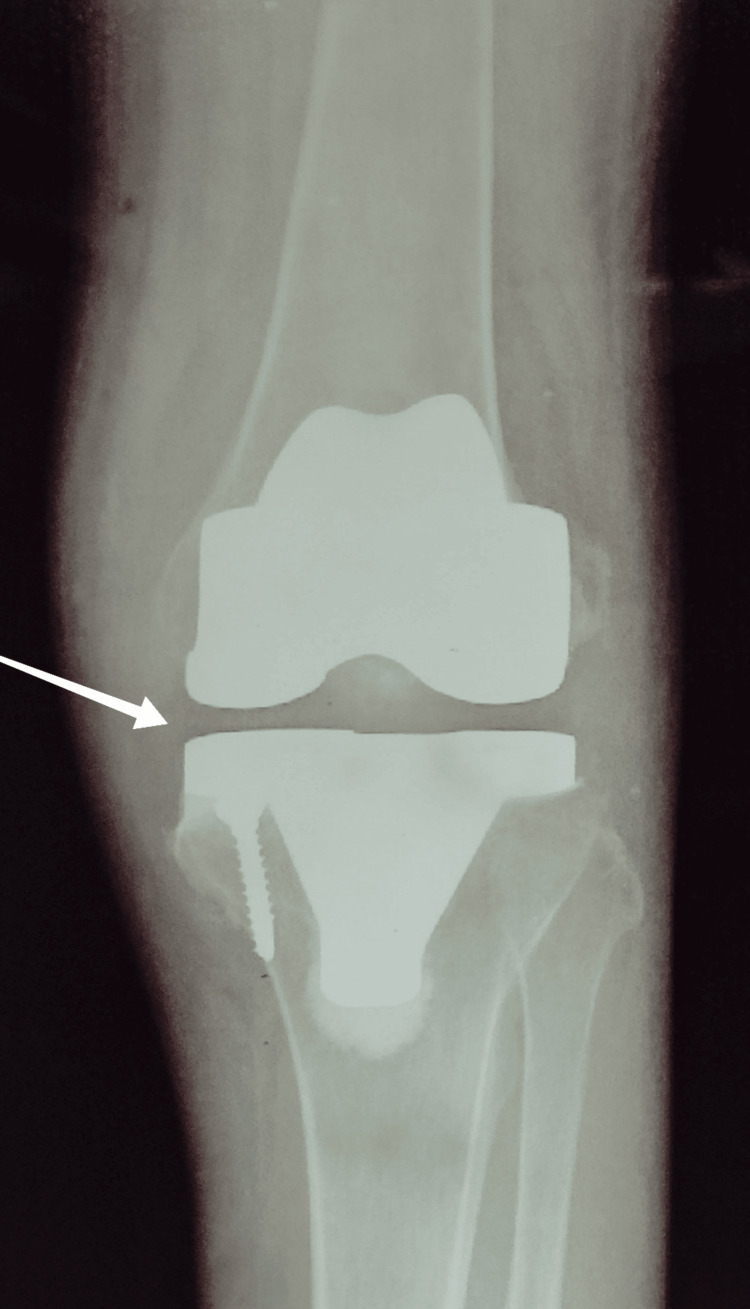
Anteroposterior X-ray view of the left knee following surgery.

To inspect the condition and replacement, radiographic scans were carried out after surgery. Figure 5 shows a pre-operative X-ray with the arrow showing the changes in the joint. In Figure 6, the left arrows symbolize the metallic implants.

Surgical procedure

TKR of the left knee was done under all aseptic precautions, cleaning, painting, and draping of the left lower leg. An approximately 12 cm incision was taken centrally over the patella's left side by a medial para-patellar sub-vastus approach. The extensor apparatus was incised medially, and the patella retracted laterally; medial release was done. Femur and tibia exposed. All the osteophytes with meniscus and ACL, PCL excised. Stability checked in flexion and extension with trial implants. The joint reduction was done. Stability was checked, and the joint was found to be stable. Sterile dressing was done, and crepe compression was applied with a long knee brace.

Physiotherapeutic intervention

**Figure 7:**
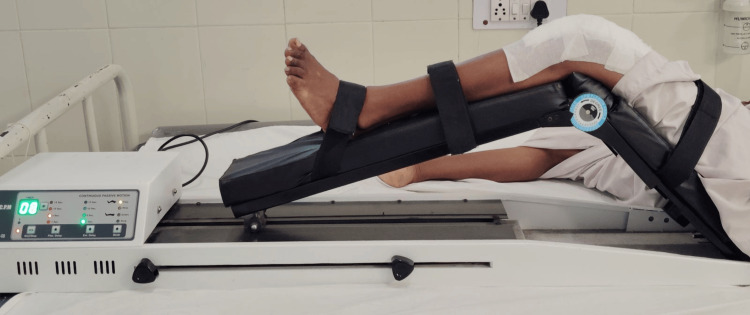
Continuous passive movement (CPM) treatment.

Table 1 shows the physiotherapy approach for TKR on the left side. Figure 7 shows continuous passive movement (CPM) treatment.

**Table 1:** Physiotherapy intervention for total knee replacement of the left knee.

Physiotherapy Rehabilitation Protocol		
Phase of rehabilitation	Goals	Intervention
During the first seven days after the operation	Patient education	The patient was explained about her condition and the benefits and value of physiotherapy. She was also explained how this treatment would improve her health by avoiding complications, thereby increasing her ability to walk, climb stairs, and do other daily activities.
	To improve the range of motion of the knee joint	To increase the joint's range of motion, the patient was taken on continuous passive movement, and by the end of the seventh day, 50 degrees of range was achieved.
	To increase the muscle strength	The quadriceps received isometric exercise, which was first gentle and rhythmic and sustained, gradual contractions, reinforced with ankle dorsiflexion, then progressed to speedy exercises. In addition, hamstring and gluteal isometric exercises were started. Assistive straight leg raises were also initiated.
	To prevent the rotation of the heel	As rotation needs to be prevented at all costs, the leg was kept elevated while maintaining a correct position using a pillow underneath.
	To gain functional independence	On postoperative day 2, the patient was ambulated (full weight bearing) with the knee brace on.
Between 7 and 14 days post-operatively	To regain the range of knee joint	During this rehabilitation period, the patient gained a range of 75 degrees on continuous passive movement (CPM), and active and active assisted range of motion exercises were given.
	To maintain the strength of the muscles	Isometrics to the quadriceps, hamstring, and gluteus were made more intensive, along with a straight leg raise with hold for 5 seconds. Also, side-lying hip abduction, dynamic quadriceps, and Vastus Medialis Oblique (VMO) strengthening were given.
	To increase functional independence	Ambulation was done in parallel bars, and stair climbing was initiated with full weight bearing and without a knee brace.

Along with the above protocol, the patient was also given some rehabilitation for the other joints that are affected by RA; this included properly supported positioning of the involved joints and correct bed posture; the limb was placed in a position of minimal discomfort, stretching was given to the involved joints and was held in the position of maximum stretch rather than applying repeated stretches, simple self-assisted passive stretching movements were given to hand and wrist.

Follow-up

After 15 days of therapeutic treatment, a follow-up was carried out. Tables 2, 3 show ROM and manual muscle testing (MMT) findings.

**Table 2:** Range of motion values prior to and following therapy.

Active Range of Motion (in degree)		
Parameter	Left lower limb - Day 1 (09/08/2023)	Left lower limb - Day 15 (23/08/2023)
Hip Flexion	0-20	0-65
Abduction	0-36	0-44
Adduction	0-22	0-38
Knee Flexion	10-25	25-80
Ankle Dorsiflexion	0-16	0-20
Plantarflexion	0-45	0-50
Eversion	0-10	0-12
Inversion	0-25	0-32

**Table 3:** Findings of manual muscle testing before and after treatment.

Manual Muscle Testing		
Parameter Manual Muscle Testing	Left lower limb - Day 1 (09/08/2023)	Left lower limb - Day 15 (23/08/2023)
Hip Flexor	3-/5	3+/5
Abductor	3/5	5/5
Adductor	3/5	5/5
Knee Flexor	2-/5	3+/5
Ankle Dorsiflexor	4/5	5/5
Plantarflexor	4/5	5/5

## Discussion

As suggested by Artz et al., rehabilitation post-TKR is strongly recommended, with a particular emphasis on physiotherapy and exercise. Physical therapy concentrates on improving mobility and obtaining functional objectives connected with discharge from the hospital throughout the hospital stay. Reeducating and functional improvement are aided by ongoing post-discharge physical therapy and exercise-based treatment [13]. In the study by Dávila Castrodad et al., many rehabilitation techniques utilized post-TKR seek to improve quadriceps strength and ROM [14]. Furthermore, these routines attempt to simplify things for people to do more challenging exercises and conduct everyday activities (ADL). Furthermore, weight-bearing biofeedback, neuromuscular electrical stimulation, and balance control appear to be beneficial additions to conventional rehabilitation [14]. Physiotherapy represents an essential element of all-encompassing treatment for those with RA along with their rehabilitation, as reported by Nogas et al [15]. Physical therapy therapies are employed at different stages of the disease based on information presented by researchers. Many essential physical therapy methods can be employed to treat RA: posture management, continuous passive movement, ROM exercises, strengthening exercises, and ambulatory training [15]. In this case, we saw the use of various therapeutic approaches like passive movements (CPM), isometric exercises, active assistive movements, and ambulatory techniques, which led to achieving a functional range of the knee and improvement in the strength of the muscle in 15 days after starting physiotherapy rehabilitation.

## Conclusions

An autoimmune condition named RA may affect individuals at any age. RA has no known cause. Its symptoms include fatigue, stiffness in joints after resting, and swelling in the affected joints. Arthritis may develop over an extended amount of time despite any symptoms. TKR is one surgical procedure for treating RA of the knee and is mainly used among others. Physiotherapy plays a crucial part in the rehabilitation process after this kind of operative procedure and in slowing the progress of RA.
